# Press needle combined with plum blossom needle improves diabetic peripheral neuropathy by inhibiting pro-inflammatory cytokines: a randomized controlled trial

**DOI:** 10.3389/fendo.2026.1956686

**Published:** 2026-09-03

**Authors:** Yongwen Zhang, Xiaoxi Nan, Lanfang Chu

**Affiliations:** 1 Department of Endocrinology, Nanjing Integrated Traditional Chinese and Western Medicine Hospital Affiliated with Nanjing University of Chinese Medicine, Nanjing, Jiangsu, China; 2 Department of Gastroenterology, Nanjing Jinling Hospital, General Hospital of Eastern Theatre Command, Nanjing, Jiangsu Province, China

**Keywords:** diabetic peripheral neuropathy, nerve fibers, plum blossom needle, press needle, pro-inflammatory cytokines

## Abstract

**Objective:**

To evaluate the clinical efficacy and safety of combined press needle and plum-blossom needle therapy in patients with diabetic peripheral neuropathy (DPN), and to explore its therapeutic mechanism related to neuroinflammation.

**Methods:**

A total of 225 DPN patients were enrolled and randomly divided into a treatment group, a control group and a sham needle group at a 1:1:1 ratio. All participants received basic treatment with oral epalrestat and mecobalamin for 6 months. The treatment group was administered press needle embedding combined with plum-blossom needle tapping; the sham needle group received identical simulated operations using blunt sham needles without effective stimulation; the control group received no additional cutaneous acupuncture interventions. Serum levels of interleukin-1β (IL-1β), interleukin-6 (IL-6) and tumor necrosis factor-α (TNF-α) were detected as primary outcomes. Secondary outcomes included motor and sensory nerve conduction velocity (NCV), nerve action potential amplitudes, modified Toronto Clinical Neuropathy Score (mTCNS) and Total Neuropathy Score (TNS).

**Results:**

After 6 months of treatment, the levels of TNF-α, IL-6 and IL-1β in the treatment group were significantly lower than those in the control group and the sham needle group (*P* < 0.001). The Cohen’s *d* values of TNF-α, IL-6 and IL-1β in the treatment group at the 2nd, 4th and 6th month were 0.753–1.364, 0.517–1.456 and 0.756–1.681 respectively, which were 1.5 to 2 times those of the other two groups. Compared with baseline, the treatment group yielded statistically greater improvements in all measured sensory conduction velocity (SCV) and motor conduction velocity (MCV) (all *P* < 0.05). Compared with the treatment group after intervention, the control and sham needle groups had significantly lower median, ulnar and sural SCV, median and ulnar MCV, ulnar sensory amplitude, ulnar, peroneal and tibial motor amplitude (all *P* < 0.05).The improvements of mTCNS and TNS in the treatment group at the 2nd, 4th and 6th month were markedly superior to those in the control and sham needle groups (*P* < 0.001).

**Conclusion:**

Combined therapy of press needles and plum blossom needles lowers pro-inflammatory cytokine levels, elevates NCV, improves DPN-associated scale scores, and ameliorates the function of both small and large nerve fibers, with favorable safety and tolerability profiles. Its therapeutic mechanism may involve inhibition of persistent neuroinflammatory cascades.

**Clinical Trial Registration:**

http://itmctr.ccebtcm.org.cn, identifier ITMCTR2025000607.

## 1 Introduction

Diabetic peripheral neuropathy (DPN) is the world’s most frequent neuropathy, affecting up to 50% of diabetes mellitus (DM) patients (1). It contributes to profound disability, diminished quality of life, and higher mortality risk (2). From a neuropathological perspective, DPN represents a distal peripheral polyneuropathy dominated by sensory manifestations, which may trigger selective degenerative damage to thinly myelinated or unmyelinated small nerve fibers, large myelinated nerve fibers, or both fiber subtypes simultaneously (3). Motor neurological deficits are characteristically absent throughout the early and intermediate disease courses, and only emerge as clinical manifestations in the terminal advanced stage of DPN progression (4). Despite its exceedingly high epidemiological prevalence and substantial cumulative clinical disease burden across diabetic populations, the standardized therapeutic regimen and comprehensive clinical management strategy for DPN remain suboptimal and unsatisfactory within routine daily clinical practice (5). Pro-inflammatory cytokines including interleukin-1β (IL-1β), interleukin-6 (IL-6) and tumor necrosis factor-α (TNF-α) mediate neuroinflammation that accelerates DPN progression by activating Nuclear Factor-κB (NF-κB) pathways, impairing Schwann cell integrity and triggering central sensory sensitization (1, 6). Longitudinal clinical evidence supports that elevated circulating levels of these cytokines predict DPN onset and severity in diabetic populations (7).

**Figure 1:**
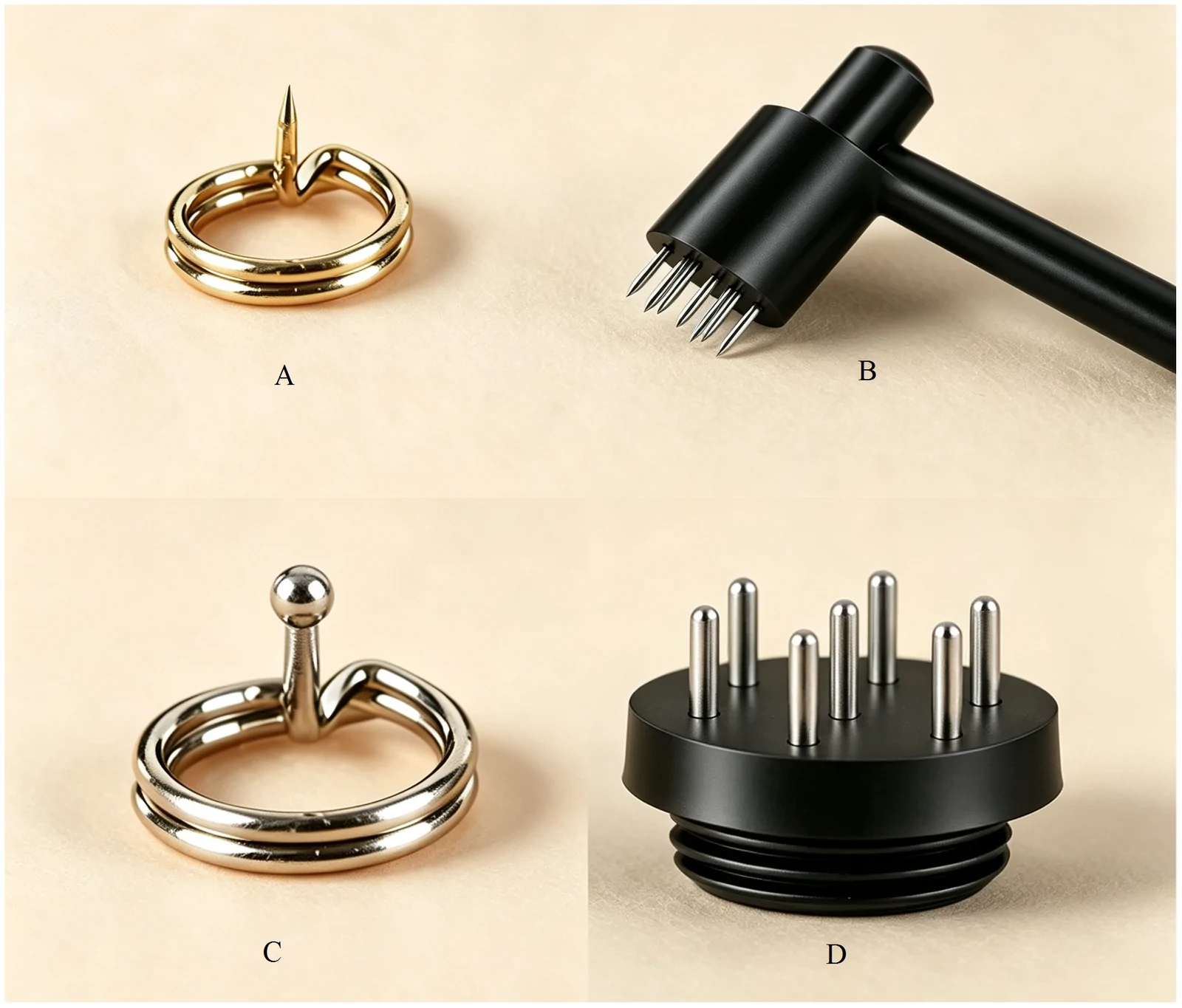
Schematic diagrams of press needle **(A)**, plum blossom needle **(B)**, sham press needle **(C)**, and sham plum blossom needle **(D)**. **(A)** Press needle is a subtype of intradermal acupuncture needle featuring an ultra-short 1–2 mm tip retained within the superficial dermis to deliver persistent mild meridian stimulation; **(B)** Plum-blossom needle is a kind of cutaneous acupuncture instrument that performs superficial percussive tapping on the skin to dredge meridians, regulate qi and blood; **(C)** The sham press needle features a closed, blunt central end without a sharp metallic tip; **(D)** For the sham plum blossom needle, all prongs are fully polished to form rounded, blunt heads.

Currently, mainstream non-pharmacological traditional Chinese medicine (TCM) interventions for DPN encompass acupuncture, warm needle acupuncture, electroacupuncture, and physical exercise regimens. Conventional acupuncture has been proven to alleviate clinical symptoms and improve nerve conduction velocity (NCV) among patients with DPN (8). The press needle is a subtype of intradermal acupuncture needle featuring an ultra-short 1–2 mm tip; secured by specialized adhesive tape, it can remain embedded at acupoints for 2 to 3 consecutive days (9) (**Figure 1A**). By contrast, the plum-blossom needle is classified as a cutaneous needle, whose therapeutic effects target the twelve supcutaneous regions of TCM theory (10) (**Figure 1B**). Relative to standard acupuncture, the press needle provides persistent, gentle stimulation at fixed acupoints. Nevertheless, its application is limited in non-acupoint cutaneous areas and regions with scant subcutaneous soft tissue. The plum-blossom needle compensates for this limitation via percussive tapping over diseased cutaneous regions, rendering it well-suited to address the erficial pathological lesions characteristic of DPN. The press needle acts on acupoints while the plum-blossom needle stimulates widespread cutaneous zones. Combined application of the two modalities achieves complementary therapeutic effects and provides full coverage of all lesion sites associated with DPN. Accordingly, the present study aims to verify the hypothesis that combined press needle and plum blossom needle therapy is both safe and clinically effective for managing DPN.

## 2 Materials and methods

This study was approved by the Ethics Committee of Nanjing Integrated Traditional Chinese and Western Medicine Hospital Affiliated with Nanjing University of Chinese Medicine (Ethics Approval No. KYLS-2025012). All subjects signed written informed consent, with guaranteed privacy protection and the right to withdraw from the trial at any time. The trial was registered on the International Traditional Medicine Clinical Trial Registry (ITMCTR, http://itmctr.ccebtcm.org.cn) with registration number ITMCTR2025000607. The trial was conducted from April to October in 2025 at the Nanjing Integrated Traditional Chinese and Western Medicine Hospital Affiliated with Nanjing University of Chinese Medicine and General Hospital of Eastern Theatre Command.

### 2.1 Participants

A total of 320 participants were initially screened and enrolled at study initiations. During the trial, 95 subjects were excluded for the following reasons: 43 patients exhibited intolerance to press needle or plum-blossom needle interventions, 40 failed to satisfy the predefined inclusion criteria, and 12 patients discontinued during the treatment phase, in the treatment group, 3 were lost to follow-up and 1 presented incomplete datasets; in the control group, 3 were lost to follow-up and 1 presented incomplete datasets; and in the sham needle group, 2 were lost to follow-up and 2 presented incomplete datasets unavailable for statistical analysis. Ultimately, 225 eligible subjects completed the study and were included in the final analytical cohort.

### 2.2 Diagnostic criteria for DPN

DPN diagnosis was validated only when all three core conditions were fulfilled:

1. Documented medical history of DM;
2. Onset of peripheral neuropathy occurred concurrently with or subsequent to DM diagnosis;
3. Clinical manifestations consistent with the typical phenotype of DPN.

### 2.3 Stratified diagnostic standards for symptomatic and asymptomatic participants

Symptomatic patients: Subjects presenting with DPN-related complaints including limb pain, numbness, paresthesia and other sensory disturbances were diagnosed with DPN if abnormalities were identified in any one of five standardized neurological assessments: ankle reflex, pinprick pain perception, vibration sense, pressure sensation, and thermal sensation.

Asymptomatic patients: DPN was confirmed when abnormal findings were detected in two or more of the above five neurological examinations.

### 2.4 Inclusion criteria

Participants were required to meet all of the following eligibility standards:

1. Aged 18–65 years, of any gender;
2. Clinically diagnosed with DPN in accordance with the above diagnostic criteria;
3. No dose adjustment or switching of medications targeting DPN within 3 months prior to trial enrollment;
4. Impaired sural nerve electrophysiological parameters: sural NCV <42 m/s, or sensory nerve action potential amplitude <6 μV;
5. Stable glycemic control satisfying all glycemic cut-off thresholds: fasting plasma glucose (FPG) ≤7.0 mmol/L, 2-hour postprandial glucose (2hPG) ≤11.1 mmol/L, glycated hemoglobin A1c (HbA1c) ≤7.0%;
6. No prior acupuncture, press needle or plum-blossom needle therapy before trial participation;
7. Voluntarily signed written informed consent prior to randomization.

### 2.5 Exclusion criteria

Any subject meeting a single item below was excluded from this trial:

1. Secondary polyneuropathy induced by non-diabetic etiologies, including chronic alcohol overconsumption, cervical/lumbar spinal lesions, chemotherapy-induced neurotoxicity, hereditary peripheral neuropathy, and other unrelated peripheral nerve disorders;
2. Confirmed diagnosis of epilepsy;
3. Coagulopathy or inherited/acquired bleeding disorders;
4. Active bacterial infection of the upper or lower extremities;
5. Women who are pregnant or breastfeeding;
6. Concurrent participation in any other interventional clinical research throughout the study period.

### 2.6 Randomization, allocation concealment and blinding

A permuted block randomization approach with a fixed block size of 6 was utilized to allocate the 225 eligible participants into three parallel study arms (treatment group, control group, and sham needle group) at a 1:1:1 ratio, with 75 subjects assigned to each group. Allocation concealment was achieved via sequentially numbered, opaque sealed envelopes. An independent third-party biostatistician generated and sealed the full randomization sequence before participant recruitment commenced. Specially trained research nurses assigned group identifiers to enrolled participants in chronological screening order by opening the sealed envelopes one by one. This trial implemented triple blinding, acupuncturists delivering interventions, trial participants, and clinical outcome assessors remained fully masked to group allocation throughout the entire research period. Acupuncture operators did not participate in outcome assessment, with complete separation between operation and evaluation teams to minimize selection and assessment bias. Secure emergency unblinding access via an interactive web-based response system was provided for scenarios involving severe adverse events or other urgent medical conditions that necessitated disclosure of participants’ allocated treatment group. If an investigator identified any unusual discomfort in a participant suspected to be associated with the assigned intervention, all trial-related assessments and procedures would be halted immediately to safeguard participant safety, in strict accordance with Good Clinical Practice (GCP) standards and institutional ethical review requirements.

### 2.7 Intervention

All enrolled patients received standardized TCM health education for diabetes formulated by our department (11), and a dedicated WeChat group was established to implement unified patient management. The research team held group communication meetings every two weeks. Prior to the initiation of interventions, blood pressure, FPG and 2hPG of all participants were well regulated, with targets maintained as follows: blood pressure <130/80 mmHg, FPG ≤7.0 mmol/L and 2hPG ≤11.1 mmol/L. Sufficient communication was conducted with each subject before treatment to alleviate anxiety and nervous emotions and improve treatment compliance. All participants were prescribed oral epalrestat combined with mecobalamin as basic medication throughout the trial. On the basis of identical baseline treatment, participants were assigned to three groups with distinct intervention regimens, and the total treatment course lasted 6 months for all subjects.

### 2.8 Treatment group

**Figure 2:**
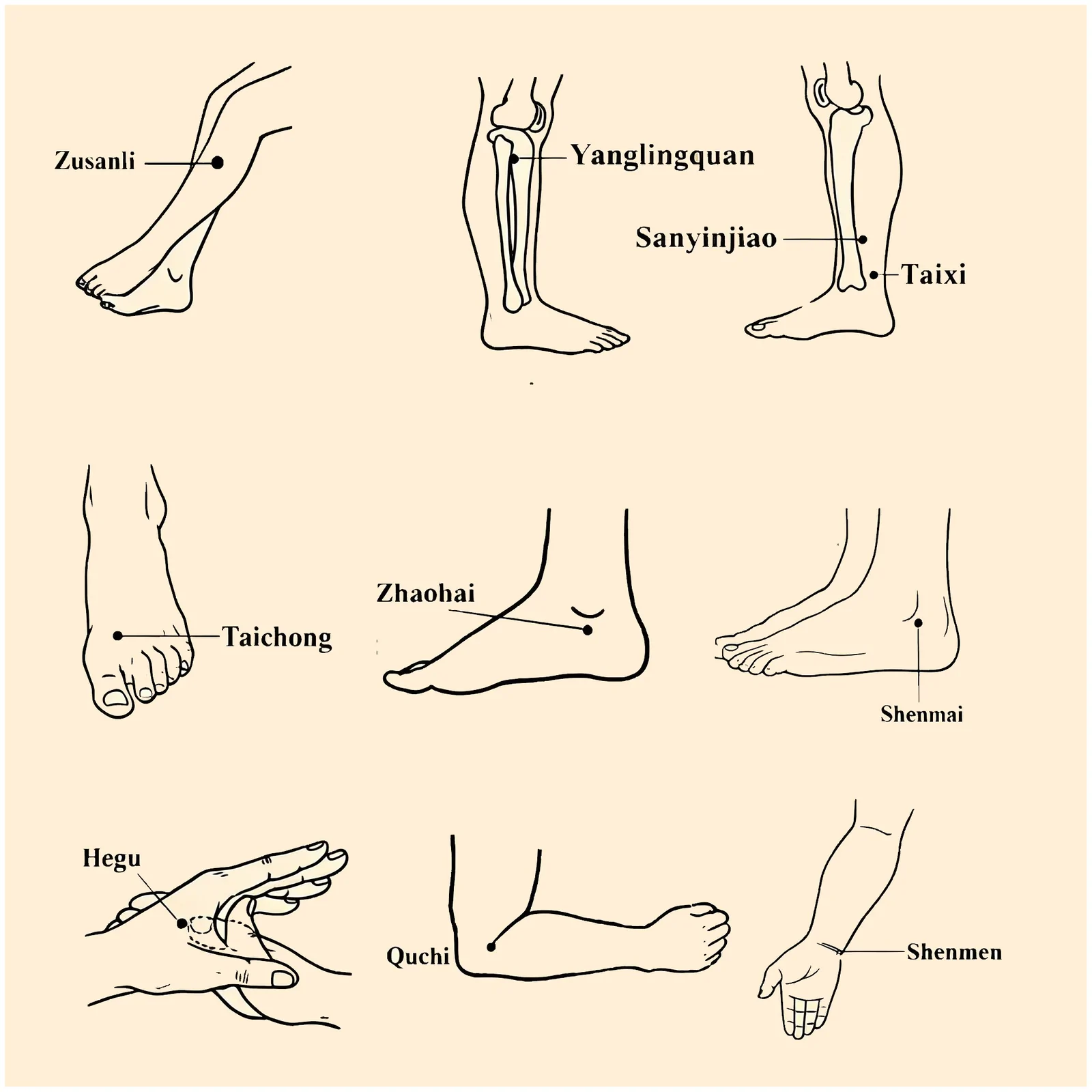
Locations of acupoints used in the present study.

Patients in this group received combined therapy of press needle embedding plus plum-blossom needle tapping. Disposable sterile stainless steel press needles (specification: 0.24 × 1.6 mm; manufactured by Beijing Luyashanchuan Medical Device Co., Ltd.) were adopted (**Figure 1A**). After routine cutaneous disinfection at acupoint sites, press needles were applied and retained for 48 hours before replacement. Plum blossom needle (manufactured by Handan Beijian Medical Devices Co., Ltd.) tapping was performed once every two days: the numbness and pain-affected areas were disinfected beforehand, followed by continuous tapping with moderate intensity for 15 minutes (**Figure 1B**). The termination criterion of tapping was defined as local cutaneous flushing accompanied by tiny pinpoint bleeding; secondary disinfection was conducted immediately after the manipulation. Bilateral acupoints were selected for intervention, including Zusanli (ST36), Sanyinjiao (SP6), Yanglingquan (GB34), Taixi (KI3), Taichong (LR3), Zhaohai (KI6), Shenmai (BL62), Hegu (LI4), Quchi (LI11) and Shenmen (HT7) (**Figure 2**).

### 2.9 Sham needle group

To ensure the integrity of blinding and eliminate non-specific placebo effects, identical acupoints, operation locations, disinfection procedures, treatment frequency, needle retention duration, single operation time and manipulation strength of operators were applied in this group, whereas no substantial meridian or cutaneous physiological stimulation was delivered.

Sham press needles: Customized placebo press needles with completely consistent appearance, patch size, color and packaging compared with genuine press needles were used (Beijing Luyashanchuan Medical Device Co., Ltd.). The center of each sham press needle was designed as a closed blunt end without sharp metal tip, which could only adhere to the epidermal surface without penetrating the skin or generating persistent subcutaneous mechanical stimulation (**Figure 1C**). Sham press needles were replaced every 48 hours with the same retention time as the treatment group.

Sham plum blossom needles: Matching sham plum-blossom needles (Handan Beijian Medical Devices Co., Ltd.) with all tips fully polished into round blunt heads were utilized (**Figure 1D**). Operators tapped the corresponding cutaneous regions for 15 minutes at the identical rhythm and force as the treatment group. The manipulation merely simulated superficial tapping sensation, without inducing cutaneous flushing, pinpoint bleeding or any cutaneous stress response.

### 2.10 Control group

Participants only received oral administration of epalrestat combined with mecobalamin, without any press needle or plum-blossom needle external therapy throughout the trial.

The treatment course lasted 6 months for subjects in all three groups.

## 3 Outcomes

### 3.1 Primary outcome

Serum concentrations of TNF-α, IL-6 and IL-1β were designated as the primary efficacy outcomes of this trial. Venous blood samples were collected at baseline (prior to enrollment), the 2nd month, 4th month and 6th month of intervention. Enzyme-linked immunosorbent assay (ELISA) kits (Wuhan Jilide Biotechnology Co., Ltd.) were adopted to test the above inflammatory biomarkers, and all experimental operations were performed in strict accordance with the manufacturer’s official instructions.

### 3.2 Secondary outcomes

#### 3.2.1 Nerve conduction velocity

Nerve conduction function was detected uniformly at baseline and the 6th month after treatment using the NeuroCare-D1 nerve conduction measuring instrument. Detected parameters included sensory conduction velocity (SCV) and sensory response amplitude of the sural nerve, ulnar nerve and median nerve, as well as motor conduction velocity (MCV) and motor response amplitude of the median nerve, ulnar nerve, peroneal nerve, and tibial nerve.

#### 3.2.2 DPN-related clinical scales

Two validated neuropathy assessment scales were evaluated at baseline, the 2nd month, 4th month and 6th month throughout the trial period: the Modified Toronto Clinical Neuropathy Score (mTCNS) and Total Neuropathy Score (TNS) (12, 13). mTCNS ranges from 0 to 19 points. This scale evaluates DPN severity by integrating subjective symptoms (numbness, abnormal paresthesia, neuropathic pain), knee and ankle tendon reflexes, vibration perception (128 Hz tuning fork test), pinprick sensation and thermal sensation. Higher scores correspond to more severe peripheral neuropathy. Stratification criteria: 0–5 points = no DPN; 6–8 points = mild neuropathy; 9–11 points = moderate neuropathy; 12–19 points = severe neuropathy. TNS spans 0–40 points. It is a multidimensional comprehensive scoring system covering subjective sensory, motor and autonomic nervous symptoms, objective clinical sensory and motor impairments, nerve conduction study (NCS) results and quantitative sensory testing findings. Elevated TNS values indicate progressive aggravation of peripheral nerve lesions.

#### 3.2.3 Safety evaluation

Adverse events (AEs) (e.g., acupoint erythema, rash, local pain, dizziness, hematoma, drug-related abnormal laboratory indicators) will be systematically documented and severity-graded in accordance with the Common Terminology Criteria for Adverse Events (CTCAE) version 5.0 (14). All participants report discomfort via the trial WeChat group. Monthly laboratory tests including routine blood, liver, renal and coagulation function are arranged. Operators record local cutaneous and systemic adverse manifestations after each intervention. The management team judges the causality of adverse events and delivers symptomatic treatment; trial intervention will be terminated promptly for severe therapy-related adverse reactions.

#### 3.2.4 Sample size

Sample size calculation was initially performed using the standard formula for two independent continuous outcomes in parallel superiority randomized controlled trials, as the primary research hypothesis focused on the between-group difference in median nerve MCV between the active treatment group and conventional control group. For a two-tailed test with α = 0.05 (Z_α/2_ = 1.96) and a statistical power of 80% (1 − β = 0.80, Z_β_= 0.84), we set the primary endpoint as median nerve MCV. Preliminary clinical pilot data yielded a baseline mean MCV of 35.02 ± 5.22 m/s (σ = 5.22). The minimal clinically meaningful intergroup difference (Δ) was predefined as 2.63 m/s between the active treatment and control groups, generating a required evaluable sample size of 62 participants per group via the two-sample formula.

This trial adopted a three-arm parallel design with equal 1:1:1 allocation (treatment group, control group, sham needle group). To maintain sufficient statistical power for the key comparison of treatment versus control, all three arms were allocated the identical sample size calculated from the primary pairwise contrast. A 20% anticipated dropout rate over the 6-month follow-up period was incorporated to adjust enrollment numbers: target sample size per arm = evaluable sample size ÷ (1 − dropout rate) = 62 ÷ 0.8 = 75 subjects per arm. In total, 225 eligible patients were recruited, with 75 participants randomly assigned to each of the three study arms.

#### 3.2.5 Statistical analysis

Statistical analyses were performed following the intention-to-treat (ITT) principle per CONSORT guidelines. All randomized participants were analyzed according to their initial allocated groups regardless of treatment adherence or loss to follow-up; multiple imputation was adopted to handle missing repeated-measure data.

Categorical variables were presented as frequencies and percentages, and between-group differences were compared by Chi-square test. Baseline continuous indicators across the three arms were analyzed using one-way analysis of variance (ANOVA), while independent-samples *t*-test was applied for two-group comparisons of normally distributed continuous data. Ordinal ranked efficacy data were compared via Kruskal–Wallis *H* test; Repeated-measures ANOVA was performed for serially measured biomarkers and nerve function indicators, including TNF-α, IL-1β, IL-6, mTCNS and TNS. The model simultaneously evaluated main group effect, main time effect, and group-by-time interaction effect. Mauchly’s test was used to examine the sphericity assumption; Greenhouse–Geisser correction was applied if sphericity was violated. Bonferroni-corrected *post-hoc* tests were used for pairwise comparisons of time points and groups. Cohen’s *d* was calculated as the standardized between-group effect size with the formula *d* = (*M*_1_−*M*_2_)/*S _pooled_*, where *S _pooled_* denotes pooled standard deviation. Per Cohen’s criteria, absolute *d* values of 0.2, 0.5, and ≥0.8 represented small, moderate, and large effect sizes, respectively, to quantify the clinical magnitude of intergroup differences. Given three planned pairwise *post-hoc* comparisons, Bonferroni correction was applied to adjust the significance level for multiple testing; the adjusted threshold for individual pairwise contrasts wasα= 0.05/3≈0.0167.All statistical tests were two-tailed, and *P* < 0.05 was defined as the threshold for statistical significance. Statistical analyses were conducted using IBM SPSS Statistics version 26.0.

## 4 Results

### 4.1 Characteristics of the participant

Baseline demographic, clinical and biochemical characteristics were comparable across the three study arms (**Table 1**). No statistically significant intergroup disparities were detected with regard to age, gender, BMI, HbA1c, FPG, 2hPG, duration of neuropathic symptoms, smoking and drinking history, total cholesterol and triglycerides (all *P* > 0.05). Hypertension and prior ischemic stroke represented the two most frequent comorbidities in the cohort. The prevalence of chronic complications and the distribution of concomitant pharmacotherapy were evenly matched among the three groups at baseline (**Table 1**).

**Table 1:** Baseline characteristics of trial participants.

Characteristics	Treatment group(n = 75)	Control group(n = 75)	Sham needle group(n = 75)	*P* value
Age mean (SD)	46.95 ± 13.59	48.10 ± 11.78	47.32 ± 11.56	0.582
Male	53 (70.68%)	54 (72.00%)	51 (68.00%)	0.862
HbA1c, %	7.24 ± 0. 45	7.29 ± 0.48	7.25 ± 0.49	0.515
Diabetes duration (years)	5.37 ± 1.60	5.78 ± 1.82	5.52 ± 1.74	0.165
FPG (mmol/l)	6.48 ± 0.49	6.51 ± 0.43	6.44 ± 0.46	0.317
2hPG (mmol/l)	9.51 ± 1.04	9.48 ± 0.92	9.62 ± 1.13	0.519
Specific DPN-related medication	36 (48.00%)	38 (50.67%)	39(52.00%)	0.881
Duration of neuropathy symptoms	4.10 ± 1.30	4.29 ± 1.32	4.33 ± 1.41	0.287
BMI (kg/m2)				
Mean (SD)	24.48 ± 3.68	25.12 ± 3.30	25.24 ± 3.53	0.199
<25, n(%)	22(29.33%)	24(32.00%)	25(33.33%)	0.863
25-30, n (%)	38(50.67%)	34(45.33%)	37(49.33%)	0.798
>30, n(%)	15(20.00%)	17(22.67%)	13(17.33%)	0.713
Smoking status, n (%)				
Never	23 (30.67%)	24(32.00%)	26(34.67%)	0.863
Former	16(21.33%)	12(16.00%)	15(20.00%)	0.689
Current	36(48.00%)	39(52.00%)	34(45.33%)	0.718
Alcohol intake, n (%)				
Daily or almost daily	16(21.33%)	14(18.67%)	15(20.00%)	0.923
Special occasions only	28(37.33%)	26(34.67%)	29(38.67%)	0.871
Never	31(41.33%)	35(46.67%)	34(45.33%)	0.795
Blood pressure, mmHg				
SBP	129.55 ± 13.54	127.25 ± 15.60	128.33 ± 14.51	0.338
DBP	84.25 ± 8.66	82.50 ± 8.04	84.21 ± 8.76	0.206
Lipids, mg/dL				
TC	249.75 ± 69.91	235.40 ± 86.86	242.37 ± 75.66	0.264
LDL-C	168.20 ± 75.33	178.90 ± 68.55	173.72 ± 72.31	0.369
HDL-C	60.25 ± 15.47	63.40 ± 18.13	63.24 ± 16.49	0.257
TG	141.75 ± 67.93	136.67 ± 59.86	143.66 ± 64.35	0.498
History of (%)				
Hypertension	30(40.00%)	32(42.67%)	29(38.67%)	0.875
Stroke	14(18.67%)	16(21.33%)	17(22.67%)	0.443
AMI	10(13.33%)	14(18.67%)	11(14.67%)	0.629
CHD Angina	18(24.00%)	16 (21.33%)	15(20.00%)	0.833
Nephropathy	12(16.00%)	10(13.33%)	13(17.33%)	0.789
mTCNS	10.32± 4.41	10.41 ± 4.23	11.06 ± 4.51	0.301
TNS	13.37 ± 5.45	12.74 ± 5.28	13.42 ± 5.61	0.441

Data are presented as mean (SD), mean and range, or frequency (percentage). n = number of participants, mTCNS: modified Toronto Clinical Neuropathy Score, TNS: Total Neuropathy Score.

## 5 Outcomes

### 5.1 Primary outcome

**Figure 3:**
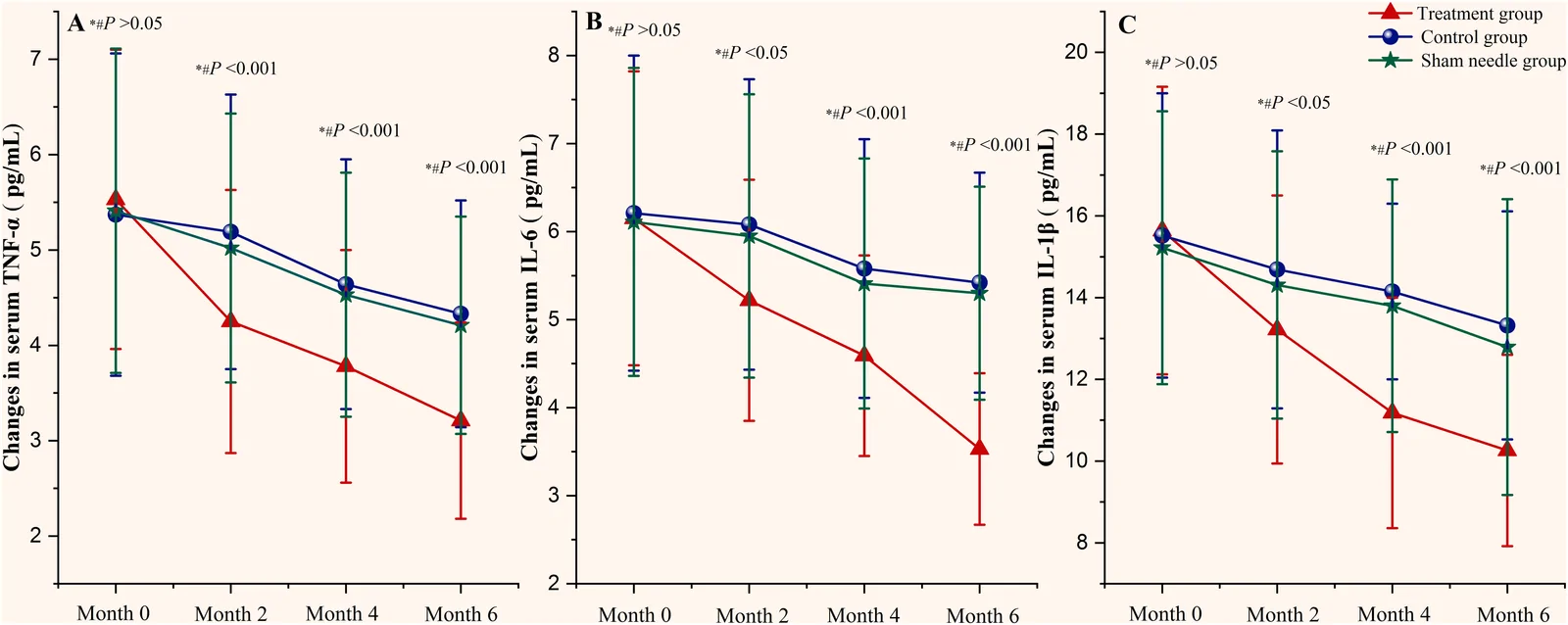
Comparison of serum pro-inflammatory cytokines levels among three groups (mean ± SD). **(A)** Serum levels of TNF-α; **(B)** Serum levels of IL-6; **(C)** Serum levels of IL-1β. ^*^vs control group; ^#^ vs sham needle group.

Before treatment, serum levels of TNF-α, IL-6 and IL-1β showed no intergroup statistical differences (*P*>0.05). Levels of the three pro-inflammatory cytokines continuously declined at the 2nd, 4th and 6th month in all three groups, with consistently lower values in the treatment group than the control and sham needle groups at all time points (*P* < 0.05). At the end of treatment, TNF-α, IL-6 and IL-1β levels in the treatment group were 3.21 ± 1.03, 3.53 ± 0.86 and 10.26 ± 2.34 respectively, which were significantly different from the control group (4.33 ± 1.19, 5.42 ± 1.25, 13.32 ± 3.79) and sham needle group (4.21 ± 1.14, 5.30 ± 1.21, 12.79 ± 3.62) (*P* < 0.001, **Figures 3A–C**).

**Figure 4:**
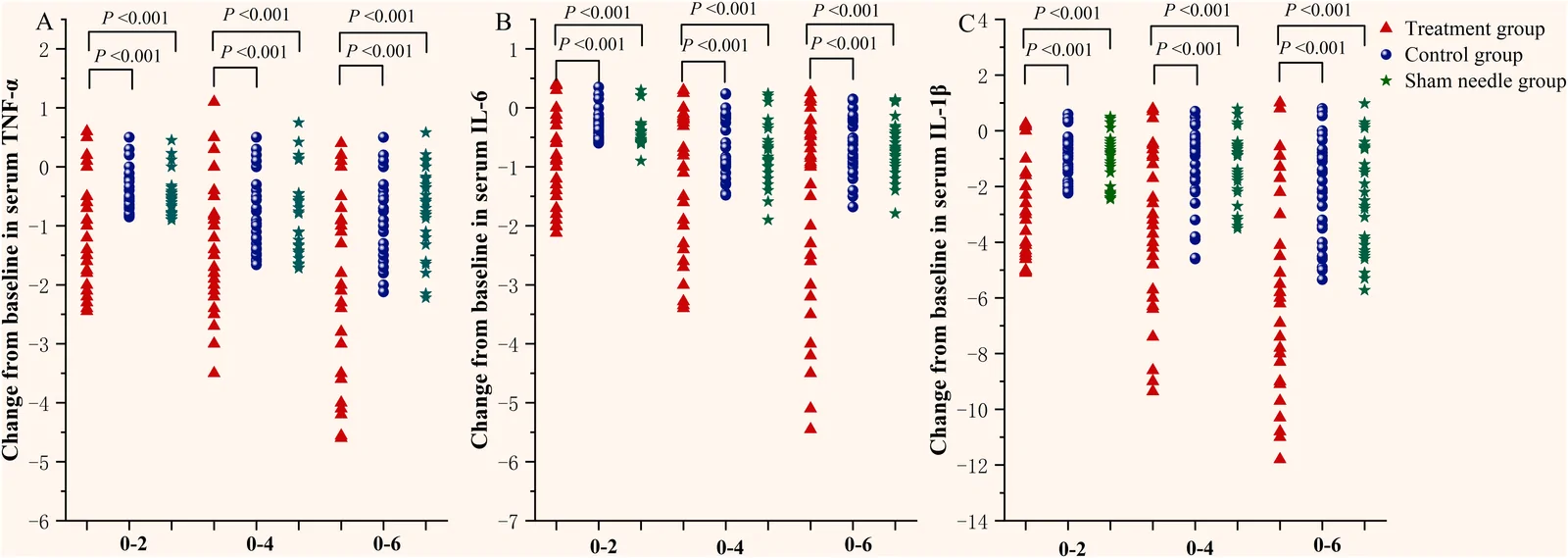
Comparison of baseline variation values of serum pro-inflammatory cytokines among three groups. **(A)** Serum levels of TNF-α; **(B)** Serum levels of IL-6; **(C)** Serum levels of IL-1β.

Baseline changes of pro-inflammatory cytokines in the three groups are shown in **Figures 4A–C**. At the 2nd, 4th and 6th month, the baseline variation values of TNF-α, IL-6 and IL-1β in the treatment group were statistically superior to those in the control and sham needle groups (*P* < 0.001). At the 6th month, the baseline change values of the three indicators in the treatment group were 2.32 ± 2.28, 2.62 ± 2.83 and 5.38 ± 6.44, with significant differences versus the control group (1.04 ± 1.08, 0.79 ± 0.94, 2.21 ± 3.17) and sham needle group (1.20 ± 1.21, 0.81 ± 0.97, 2.43 ± 3.31) (*P* < 0.001).

### 5.2 Secondary outcomes

**Figure 5:**
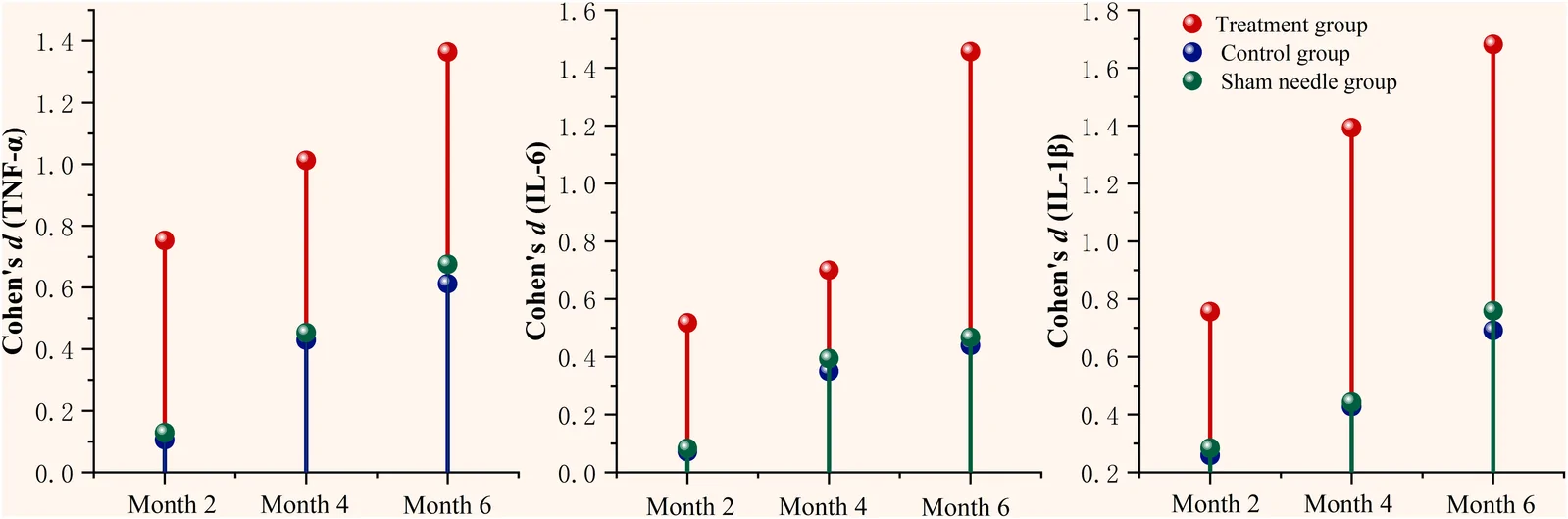
Cohen’s *d* values of serum pro-inflammatory cytokines in three groups.

Cohen’s *d* effect sizes for intergroup comparisons across the three study arms are presented in **Figures 5A–C**. For TNF-α, IL-6, and IL-1β measured at the 2-, 4-, and 6-month follow-up visits, the ranges of Cohen’s *d* in the treatment group were 0.753–1.364, 0.517–1.456, and 0.756–1.681, respectively. Corresponding ranges were 0.106–0.612, 0.072–0.439, and 0.259–0.691 for the control group, and 0.129–0.676, 0.083–0.467, and 0.284–0.759 for the sham needle group. At the 6-month endpoint, all three cytokines yielded large effect sizes (Cohen’s *d* ≥ 0.8) within the treatment group, with Cohen’s *d* values 1.5 to 2 times greater than those observed in the control and sham needle groups.

In SCN, all three measured nerves of the treatment group increased significantly (all *P* < 0.001). The control and sham needle groups only had obvious improvements in median and ulnar sensory nerves (*P* < 0.05), without statistical changes in sural nerve velocity. For MCN, all four motor nerves improved markedly in the treatment group (*P* < 0.05). Only median motor velocity increased significantly in the control group (*P* < 0.05), while the sham needle group showed improvements in median and tibial motor nerves (*P* < 0.05). Intergroup differences in these velocity changes were significant (*P* < 0.001, **Table 2**). In sensory nerve amplitude, the treatment group had elevated median and ulnar amplitudes (*P* < 0.001). Only median sensory amplitude increased in the other two groups (*P* < 0.05). Intergroup differences were significant for median (*P* = 0.035) and sural amplitudes (*P* = 0.002). All motor nerve amplitudes were significantly elevated in the treatment group (*P* < 0.05). Both control and sham groups merely exhibited higher ulnar motor amplitude (*P* < 0.001). Compared with the treatment group after intervention, the control and sham needle groups had significantly lower median, ulnar and sural SCV, median and ulnar MCV, ulnar sensory amplitude, ulnar, peroneal and tibial motor amplitude (*P* < 0.05, **Table 2**).

**Table 2:** Change in nerve conduction velocity and amplitude comparing baseline and month 6.

Nerve	Treatment group (n = 75)			Control group (n = 75)					Sham needle group(n = 75)			*P*-value for change
	n	Baseline	Month 6	n	Baseline	Month 6			n	Baseline	Month 6	
Nerve conduction velocity												
Sensory nerves (m/s)												
Median	71	35.43 ± 4.51	44.38 ± 5.73^**^	67	36.13 ± 4.43		38.48 ± 4.75^*##^	70		36.35 ± 4.61	38.89 ± 4.84^*##^	<0.001
Ulnar	67	36.63 ± 5.43	43.46 ± 6.62^**^	62	37.15 ± 5.53		39.26 ± 6.24^*##^	65		36.53 ± 5.64	39.82 ± 6.53^*#^	<0.001
Sural	62	37.37 ± 5.27	45.12 ± 6.42^**^	60	38.01 ± 5.45		39.41 ± 5.62^##^	59		37.84 ± 5.51	39.82 ± 5.89^##^	<0.001
Motor nerves (m/s)												
Median	54	38.21 ± 4.46	44.62 ± 4.52^**^	55	38.48 ± 4.61		40.38 ± 4.87^*##^	57		38.75 ± 4.66	41.22 ± 5.07^*##^	<0.001
Ulnar	56	41.45 ± 4.87	47.68 ± 5.35^**^	58	42.16 ± 4.46		43.58 ± 5.17^##^	54		41.74 ± 4.58	43.21 ± 5.26^##^	<0.001
Peroneal	62	37.63 ± 5.48	40.48 ± 5.67^*^	64	38.36 ± 5.17		39.15 ± 5.43	61		37.85 ± 5.41	39.57 ± 5.64	<0.001
Tibial	59	39.29 ± 5.21	44.47 ± 5.58^**^	61	38.97 ± 5.62		40.36 ± 5.98	57		39.36 ± 5.46	41.58 ± 6.15^*^	<0.001
Response amplitude												
Sensory nerves (uV)												
Median	60	5.32 ± 2.24	7.67 ± 3.34^**^	59	5.39 ± 2.53		6.64 ± 2.69^*^	63		5.53 ± 2.61	6.78 ± 2.76^*^	0.035
Ulnar	54	5.46 ± 2.58	7.74 ± 2.97^**^	52	5.18 ± 2.74		6.25 ± 3.34^#^	56		5.36 ± 2.81	6.51 ± 3.47^#^	0.060
Sural	51	4.58 ± 2.42	5.37 ± 2.69	54	4.68 ± 2.48		5.15 ± 2.57	55		4.71 ± 2.52	5.28 ± 2.84	0.002
Motor nerves (mV)												
Median	55	3.86 ± 2.65	5.69 ± 2.90^*^	57	3.74 ± 2.07		4.16 ± 2.35	54		3.91 ± 2.24	4.49 ± 2.62	0.012
Ulnar	62	2.82 ± 1.74	5.83 ± 2.54^**^	61	2.69 ± 1.53		3.86 ± 1.98^**##^	64		2.77 ± 1.47	3.98 ± 1.85^**##^	<0.001
Peroneal	52	3.58 ± 2.42	6.57 ± 3.73^**^	54	3.76 ± 2.53		4.35 ± 2.81^##^	55		3.63 ± 2.62	4.62 ± 2.89^#^	0.001
Tibial	58	2.68 ± 1.79	4.53 ± 2.18^**^	57	2.84 ± 1.68		3.35 ± 1.79^#^	60		2.73 ± 1.75	3.31 ± 1.85^#^	0.004

Compared with baseline, ^*^P < 0.05, ^**^P < 0.001; Compared with treatment group after intervention, ^#^*P* < 0.05, ^##^*P* < 0.001.

**Figure 6:**
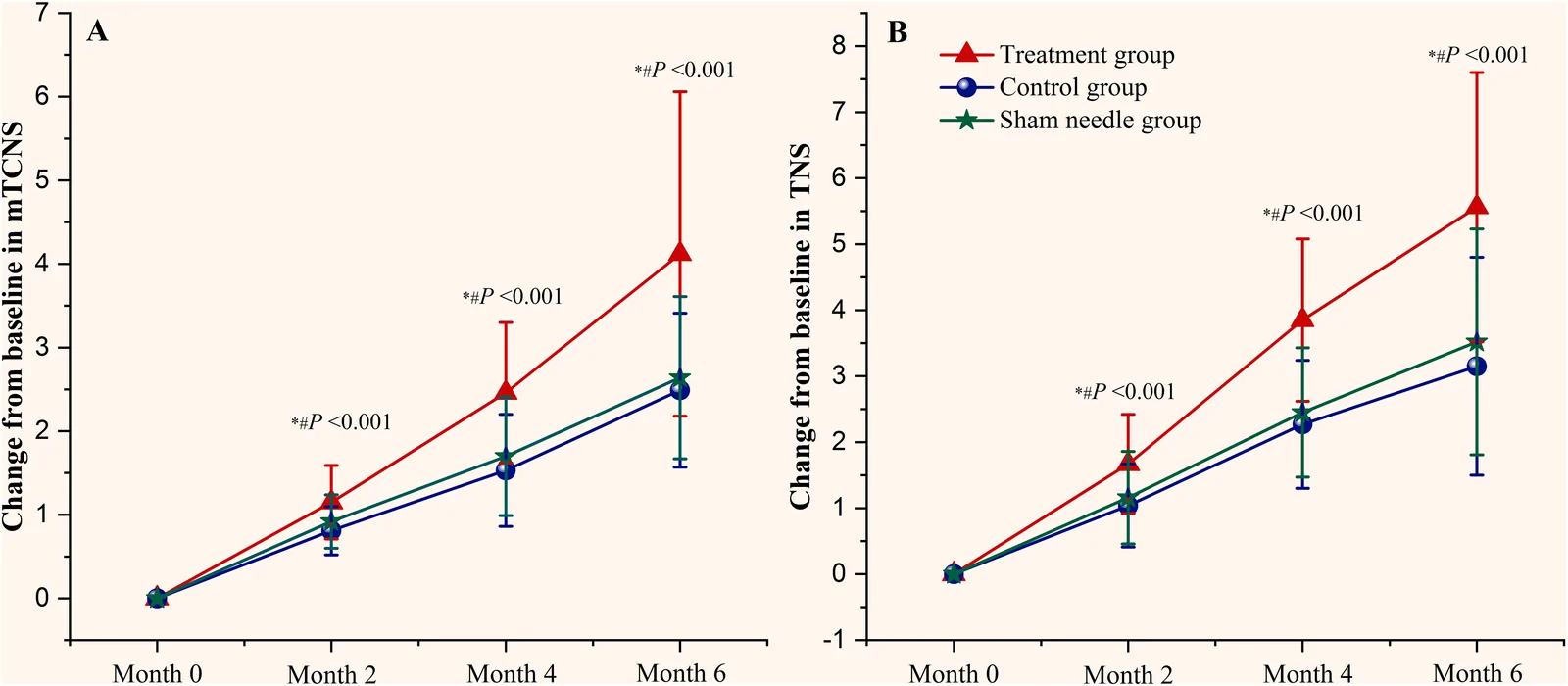
Comparison of baseline variation values of mTCNS and TNS among three groups (mean ± SD). **(A)** DPN-related clinical Scale: mTCNS; **(B)** DPN-related clinical Scale: TNS. ^*^vs control group; ^#^vs sham needle group.

After 6 months of treatment, the baseline improvement values of mTCNS and TNS continuously increased in all three groups, with larger improvement amplitude prolonged with follow-up duration (**Figure 6**). Intergroup comparison revealed that the mTCNS and TNS improvements of the treatment group at the 2nd, 4th and 6th month were significantly better than the control and sham needle groups (*P*<0.001). At trial termination, the baseline variation of mTCNS in the treatment group was 4.12 ± 1.94, statistically higher than the control group (2.49 ± 0.92) and sham needle group (2.64 ± 0.97) (*P* < 0.001). The baseline variation of TNS in the treatment group was 5.36 ± 2.04, markedly superior to the control group (3.15 ± 1.65) and sham needle group (3.52 ± 1.71) (*P* < 0.001, **Figure 6**).

### 5.3 Adverse events

A total of six mild AEs were recorded during the entire trial period, consisting of transient limb paresthesia, transient local acupuncture-site pain, and pruritus at the patch attachment sites. Among these adverse events, four cases (5.33%) occurred in the treatment group and two cases (2.67%) in the sham acupuncture group; the intergroup difference in AEs incidence was not statistically significant (*P* = 0.405). All adverse symptoms resolved completely following symptomatic supportive care, and no participants discontinued trial participation owing to adverse reactions.

## 6 Discussion

Findings from the present study demonstrate that combined press needle and plum blossom needle treatment decreases pro-inflammatory cytokine levels, enhances NCV, relieves clinical manifestations reflected by DPN-relevant scale scores, and improves the function of both small and large nerve fibers. This intervention exhibits good safety and tolerability, and its therapeutic effects may be mediated through inhibition of persistent neuroinflammatory cascades. A recent global meta-analysis demonstrated that the prevalence of DPN in T2DM patients (31.5%, 95%CI 24.4–38.6%) is higher than that in T1DM patients (17.5%, 95%CI 13.1–36.5%) (15). As a prevalent complication of DM, DPN still lacks satisfactory therapeutic approaches, making exploration of effective interventions an urgent task (16). Acupuncture has been widely adopted for DPN treatment with confirmed efficacy, recognized by the American Pain Society and the National Center for Complementary and Alternative Medicine (17). Further research on TCM etiology and pathogenesis of DPN is required to develop mechanism-based TCM therapies for symptom relief and improved patient prognosis (18). Press needle and plum blossom needle act superficially on cutaneous regions, consistent with the core pathogenesis of DPN. The press needle delivers sustained mild stimulation at acupoints with its superior 2–3 day retention capability compared with conventional acupuncture, while the plum blossom needle taps extra-acupoint cutaneous regions to induce pinpoint bleeding and discharge internal pathogens. The two therapies complement and reinforce each other.

Neuroinflammation has emerged as a key contributor to the pathogenesis of DPN (19). Chronic low-grade inflammation mediated by IL-1β, IL-6 and TNF-α acts as a core pathogenic driver of DPN, independent of sustained hyperglycemia alone. Hyperglycemia and lipotoxicity trigger NF-κB and NLRP3 inflammasome cascades in peripheral nerves, dorsal root ganglia and circulating immune cells, which robustly upregulate the secretion of the pro-inflammatory cytokines (20–22). TNF-α and IL-1β primarily induce Schwann cell dysfunction, myelin sheath breakdown and microvascular hypoperfusion within peripheral nerves, directly slowing motor and sensory nerve conduction velocities; IL-6 further amplifies spinal central sensitization and persistent neuropathic hyperalgesia, linking peripheral nerve lesions to central nervous system remodeling in complicated DPN cases (23). Large prospective clinical cohorts confirmed that elevated baseline plasma TNF-α, IL-6 and IL-1β serve as independent predictors for the 5-year incidence and progressive severity of DPN among T2DM individuals, even after adjusting for HbA1c, disease duration and lipid profiles (7).

In patients with DPN, small-fiber lesions induce positive sensory symptoms such as nocturnal lower-limb pain, hyperalgesia, allodynia and paresthesia, with motor impairment developing only in late-stage disease (24). Large-fiber neuropathy presents as negative symptoms including foot numbness and impaired balance, which are often unrecognized until neurological assessment. Most DPN patients display combined small- and large-fiber dysfunction (25). After 6 months of intervention, serum TNF-α, IL-6 and IL-1β were markedly reduced in the treatment group, with significantly better outcomes than the control and sham needle groups (all *P* < 0.001). All corresponding Cohen’s *d* values indicated large effect sizes (*d* ≥ 0.8), 1.5–2-fold higher than those in the two control arms. Reduced pro-inflammatory cytokines correlate with functional recovery of both small and large nerve fibers in DPN. Suppression of persistent neuroinflammatory cascades serves as a key therapeutic target in this trial. Lower cytokine levels were associated with preserved axonal density, mitigated demyelination and improved NCV (20). Our results suggest that combined press needle and plum blossom needle may alleviate DPN manifestations via the downregulation of inflammatory cytokine production; nevertheless, the exact underlying molecular mechanisms have not yet been fully clarified.

A proportion of patients with DPN present no obvious clinical symptoms. Therefore, DPN scales and NCV measurements were adopted as two complementary evaluation tools to comprehensively assess the therapeutic efficacy of combined press needle and plum blossom needle therapy. Following the 6-month intervention, the treatment group exhibited significantly greater reductions from baseline in mTCNS and TNS at all follow-up time points up to the 6-month endpoint. Compared with the control and sham groups, the treatment group achieved significant improvements in most measured sensory and motor NCVs and response amplitudes. NCV mainly reflects the function of large myelinated Aα/Aβ nerve fibers. Reduced NCV is driven by demyelination or the loss of large nerve fibers, while elevated NCV signals remyelination and restored large-fiber function, serving as a core electrophysiological biomarker for large-fiber nerve repair (26). Improved nerve response amplitudes reflect preserved axonal integrity and functional recovery, which correspond to the reversal of early diabetic nerve injury, restored sensory and motor performance, and a decreased risk of numbness, muscular weakness and diabetic foot ulcers (27). Concurrent improvements in clinical scale scores and NCV measurements reveal that this combined needle therapy facilitates remyelination and axonal repair of large nerve fibers to relieve DPN symptoms and reduce the risk of diabetic foot lesions.

The strengths of this study include recommending simple, safe and easy-to-perform press needles and plum-blossom needles for the treatment of DPN, as well as exploring its therapeutic mechanisms from the perspective of neuroinflammation. Limitations of this trial include limited sample size and short 6-month observation duration. Multi-center long-term clinical trials and animal experiments are required to verify long-term efficacy and elaborate the molecular mechanisms of the two cutaneous needle therapies for DPN. Future research directions include establishing standardized operation protocols for combined press needle and plum blossom needle therapy, and constructing an integrated standardized TCM diagnosis and treatment platform integrating acupoint application, herbal fumigation, acupoint injection and cutaneous acupuncture for DPN management.

In summary, combined therapy of press needles and plum-blossom needles lowers pro-inflammatory cytokine levels, elevates NCV, improves DPN-associated scale scores, and ameliorates the function of both small and large nerve fibers, with favorable safety and tolerability profiles. Its therapeutic mechanism may involve inhibition of persistent neuroinflammatory cascades.

## Data Availability

The raw data supporting the conclusions of this article will be made available by the authors, without undue reservation.
